# How Can Molecules Induce Hemorrhoids? The Role of Genetics and Epigenetics in Hemorrhoidal Disease

**DOI:** 10.3390/ijms26199394

**Published:** 2025-09-26

**Authors:** Barbara Parol, Oliwia Sas, Mateusz Mazurek, Krzysztof Data, Slawomir Wozniak, Zygmunt Domagala

**Affiliations:** 1 Clinical and Dissecting Anatomy Students’ Scientific Club, Wroclaw Medical University, 50368 Wroclaw, Poland; oliwia.sas@student.umw.edu.pl (O.S.); mateusz.mazurek@student.umw.edu.pl (M.M.); 2 Division of Anatomy, Department of Human Morphology and Embryology, Wroclaw Medical University, 50368 Wroclaw, Poland; krzysztof.data@umw.edu.pl (K.D.); slawomir.wozniak@umw.edu.pl (S.W.); zygmunt.domagala@umw.edu.pl (Z.D.); 3 Walbrzych Branch, Wroclaw Medical University, 58300 Walbrzych, Poland

**Keywords:** hemorrhoids, genetics, epigenetics, gene expression, ncRNA, microRNA, miRNA, inflammation, targeted therapy, molecular therapy

## Abstract

The pathophysiology of hemorrhoids remains elusive; however, recent research has increasingly focused on the role of genetic and epigenetic mechanisms in this condition, offering prospects for targeted treatments. We conducted a review using PubMed, Embase, and Google Scholar, supplemented by citation searching, to summarize the current knowledge of the involvement of genetic and epigenetic mechanisms in hemorrhoids. Our review of 250 papers suggests that several genes, including *FOXC2*, *NOX*, *NOS*, and *CALM3*, may be responsible for the predisposing changes leading to the development of hemorrhoids. These genes have been associated with varicose veins, inflammation, and connective tissue alterations. Additionally, epigenetic mechanisms, particularly those mediated by miRNA, have been implicated in the clinical symptoms of hemorrhoids. While epigenetic regulation may influence inflammation, dilated vessels, and connective tissue degeneration, the exact mechanisms involved in these processes remain unclear. Furthermore, certain predisposing factors for hemorrhoids appear to involve both genetic and epigenetic mechanisms. This knowledge contributes to a better understanding of hemorrhoids and holds promise for developing novel therapeutic approaches.

## 1. Introduction

Around the anus and the lower rectum, anal cushions are located, which consist of blood vessels and non-vascular components: epithelium (mucosa or anoderm), connective tissue (elastic and collagenous), a layer of muscular tissue (named the muscle of Treitz), and an anchoring system which connects them to surrounding tissue [1,2]. In some cases, leading to pathological changes, they can become swollen, inflamed, and displaced, which results in the development of pathological hemorrhoids, in other words, hemorrhoidal disease [1,2,3,4,5,6,7,8].

Currently, there are three main theories regarding the pathogenesis of hemorrhoids: the varicose vein theory, where hemorrhoids are supposed to originate from abnormally dilated and congested veins of the submucous internal rectal venous plexus [2,8,9,10,11,12]; the vascular hyperplasia theory, where the hemorrhoids develop from the hyperplasia of the corpus cavernosum recti [2,13]; and the sliding anal lining theory, which implies that hemorrhoids are a result of the degradation of the surrounding connective tissue that causes normal anal cushions to displace, possibly because of an increase in the intra-abdominal pressure, caused by constipation and prolonged straining [2,8,9,10,11,12].

The exact pathophysiology of hemorrhoids remains elusive, as there are no non-ambiguous, well-proved theories. The role of genetics and epigenetics is currently suspected and studied; that is why it seems important to pose a crucial question about the role of those factors in the development of hemorrhoidal disease and summarize and review the current knowledge in this field, in the hopes of deepening our knowledge about this condition. Our review aims to describe the molecular mechanisms that influence the development of hemorrhoidal disease.

Hemorrhoids are the most commonly occurring proctological disease, and it is estimated that every fourth person suffers from this condition. Because of the scale of the problem, it is a major medical issue—data from the National Center for Health Statistics suggest that approximately 10 million people in the United States suffer from hemorrhoids [7], with an annual visit count higher than for colon cancer, irritable bowel syndrome, inflammatory bowel disease, ulcerative colitis, or Crohn’s disease [14]. With this many cases, new ways of treatment are constantly in demand [15]. While reviewing the gathered studies, we have come across various molecular processes that take part in the development and progression of hemorrhoids. Most, if not all, of these mechanisms seem to be a potential base for finding new targeted molecular treatments for hemorrhoids. Targeted therapy based on the molecular processes that occur in diseases is not a new concept—numerous works that report on new methods of treatment based on the discovered molecular characteristics of a particular disease have been published: in use are cell-free DNA [16], mRNA decay inhibitors [17,18], and anti-cytokines [19]. Some of those methods are based on mechanisms that are similar or corresponding to those uncovered in hemorrhoids—inflammatory bowel disease can be treated with anti-cytokine drugs, targeted at cytokines overexpressed in this condition [19], influencing COX2 (cyclooxygenase-2) and PGE2 (prostaglandin E2) expression to mitigate the inflammation. After research and some possibly needed modifications, those mechanisms—or similar ones—could be used to treat hemorrhoids as well. However, extensive expertise in the subject is required to develop such a treatment, and in most cases, our current understanding is not sufficient—it seems quintessential to regularly compile the current information about known conditions to accelerate and maximize the potential of treatment-related research.

## 2. Why Do Hemorrhoids Develop?

So far, multiple factors have been claimed to be involved in hemorrhoidal development, especially states that cause an increase in the intra-abdominal pressure: prolonged straining during constipation, obesity, and pregnancy. These also include internal agents such as genetic and immune factors, aging, pelvic floor dysfunction, chronic diarrhea, and environmental agents—diet, sedentary lifestyle, hygiene, alcohol consumption, diverse sexual behaviors (e.g., anoreceptive intercourse), and prolonged sitting [7,8,9,10,12,20,21,22].

Those factors cause stress to tissues, which respond with an inflammatory reaction manifested by vein dilation, the destruction of the extracellular matrix of the hemorrhoidal supporting tissue together with anal subepithelial muscle [7], and, in the end, the promotion of angiogenic and proliferative factors [23,24], which is in line with all three main theories regarding the development of hemorrhoids: the varicose vein theory, the vascular hyperplasia theory, and the sliding anal lining theory. Occurring activities are regulated by, among others, epigenetic mechanisms, such as non-coding RNA (ncRNA) [22,25,26,27,28]. They function as a messenger when the stress signal occurs and regulate intracellular pathways to trigger a response to that signal. In the case of a deficiency in ncRNAs, a response to a particular stress signal is greatly weakened [9,29]. NcRNAs are engaged in regulating expression by engaging in splicing and translation and are a critical factor in the post-transcriptional regulation of transcriptome expression [28]. The following belong to the group of ncRNAs: long non-coding RNA (lncRNA), microRNA (miRNA), which is particularly important in hemorrhoids, circular RNA, ribosomal RNA (rRNA), transfer RNA (tRNA), and small nuclear RNA (snRNA) [30].

MiRNAs are small, single-stranded, non-coding RNA particles that can regulate gene transcription—they bind to the 3′ untranslated region (3′-UTR) of the mRNA molecule transcribed from the target gene, which causes inhibition and induces cleavage of the mRNA by dicer enzymes and silencing the gene expression [31,32,33,34,35,36,37]. One molecule of miRNA can target hundreds of genes, and one gene can be targeted by multiple miRNAs, forming a network that can regulate many pathways. The identification of genes crucial in the development of hemorrhoids is a subject of research, and some of these genes remain to be uncovered [38,39,40,41,42,43].

Below, we discuss the currently discovered and documented processes regarding the molecular mechanisms occurring in hemorrhoids, with their proven or theorized mechanisms of regulation, in the hopes of systematizing our knowledge.

## 3. Inflammation

In hemorrhoidal tissue, we can observe very intense inflammation, involving both the dense vascular system and the supportive connective tissue. Inflammation can lead to the pathological changes we can observe in hemorrhoids: mucosal ulceration, ischemia, thrombosis, further vessel dilation, and the distortion of the smooth muscle layer, together with the surrounding supportive tissue [7,20,21]. This process occurs with the activation of T-lymphocytes, macrophages, neutrophils, monocytes, mast cells, and dendritic cells [44,45], which are responsible for secreting pro- and anti-inflammatory cytokines. The balance, however, shifts very soon to the dominance of pro-inflammatory cytokines, which are highly expressed in hemorrhoids. Among them, there are RANTES (Regulated on Activation, Normal T-cell Expressed and Secreted, CCL-5), TNF-α, and VEGF (vascular endothelial growth factor), which, in addition to proangiogenic function, also increase cell permeability, and as a result, contribute to the inflammation process [45,46,47,48], as well as interleukines IL-1β, IL-6, IL-8, IL-17, and interferon gamma (IFN-γ)—these cytokines were found to be significantly overexpressed in hemorrhoids. IL-10, an anti-inflammatory cytokine, is underexpressed [49] (see Table 1). A study has shown that some substances (e.g., polyherbal formulation Anoac-H, turmocin) help to downregulate the expression of those cytokines in fibroblasts and macrophages of hemorrhoid tissues, which leads to a reduction in inflammation, and, due to VEGF’s primary function, to a decrease in vascular density, which is increased in hemorrhoids [44,50].

In addition to that, genes of CGRP (calcitonin gene-related peptide) and substance P (SP), together with TRPV1 (transient receptor potential cation channel, vanilloid receptor 1), which are involved in regulating the expression of cytokines in blood cells [51,52,53], were found to take part in regulating that expression in serum in the case of hemorrhoids as well [54]. Some substances were proven to inhibit the secretion of pro-inflammatory cytokines in serum via downregulating those genes, which helped to ease hemorrhoid symptoms, suggesting that those genes are upregulated in hemorrhoids and take part in their development.

Other than those factors, increased levels of COX-2 were detected in hemorrhoidal tissues, and they were secreted by fibroblasts and macrophages [23,49,55]. COX-2 is a molecule involved in the production of prostaglandins and main pain mediators, as well as supporting the inflammation process. It is likely to be the cause of pain in hemorrhoid disease [44,56].

Zhou et al. have found increased miR-770 levels in hemorrhoid tissues. This miRNA promotes the polarization of MΦ1 macrophages, which results in an increased observed infiltration of these macrophages in hemorrhoid-affected tissues. MiR-770 suppresses RYBP, a member of the PRC1 complex—a complex responsible for inhibiting gene expression and thus weakens the transcriptional repression. A result of this process is the upregulation of genes encoding inflammatory compounds like NF-κB (nuclear factor-κB-dependent), IL-1β, and TNF-α, further fueling the inflammation [57].

In summary, pro-inflammatory cytokines, especially VEGF, are more expressed in hemorrhoids than in normal cells and play an important part in their pathogenesis, mainly angiogenesis, and maintaining the inflammatory state, which leads to progression of the disease and clinical symptoms.

## 4. The Role of Vesicles

Some studies regarding the role of miRNA in hemorrhoids found that miRNAs were upregulated or downregulated in hemorrhoids and are possibly targeting genes, based on available databases, involved in various vesicular pathways (see Table 2) [22,25].

According to Song et al., the miRNAs upregulated in hemorrhoid tissues were miR-375, miR-215-5p, miR-192-5p, miR-143-3p, miR-187-3p, miR-194-5p, miR-145-5p, miR-490-3p, and miR-145-3p, while the downregulated miRNAs in hemorrhoids were miR-376b-3p, miR-34a-5p, miR-152-3p, let-7c-5p, miR-107, miR-517a-3p, miR-517b-3p, miR-1307-5p, miR-190a-5p, miR-378a-5p, miR-708-3p, miR-450a-5p, miR-450a-5p, miR-30e-5p, and miR-532-5p. Two of these, miRNA-133b and miRNA-133a-3p, shared 32 of the possible target genes, and the rest of the miRNAs had independent potential target genes [25].

The genes possibly targeted by them, for both upregulated and downregulated miRNAs, are involved mainly in cell composition and protein binding. The upregulated miRNAs’ most prominent target, with the most changes in gene expression, is the endocytosis pathway, and the downregulated miRNAs focus the most on genes belonging to the synaptic vesicle pathway. This leads to the higher expression of endocytosis pathway genes and the lower expression of synaptic vesicle pathway genes, which was identifiable in tests and suggests that the development of hemorrhoids may be linked to an imbalance between the expression of the endocytosis and synaptic vesicle cycle pathway genes, generated by changes in the levels of miRNAs in hemorrhoidal cells. Those two pathways in hemorrhoidal tissue might be responsible for regulating the infiltration of inflammatory cells, the proliferation of vascular endothelial cells, the edema of interstitial cells, or other processes, although that is still a subject for further research [25].

Differences in miRNA expression were found in extracellular vesicles as well [22]. Extracellular vesicles are membranous structures originating from cell membranes [58]. They have a role in cancer progressionmetastasis [59], wound healing [60], angiogenesis [61] and immunoregulation [62]. They also function as messengers between cells [63], carrying proteins, lipids, and RNAs [64,65]. Hemorrhoidal extracellular vesicles were found to be carrying different molecules than vesicles derived from healthy tissues, with the most difference being in the content of miRNAs. There were 245 upregulated miRNAs found in hemorrhoidal extracellular vesicles, and within them, the 10 most prominent were miR-6741-3p, miR-6834-3p, miR-4254, miR-6804-3p, miR-744-3p, miR-8485, miR-299-5p, miR-4636, miR-3175, and miR-4658. Those miRNAs seem to influence the transcription process and target genes involved in protein kinase activity, transcriptional activity, and ubiquitin–protein function, and are most active in proximity to cell junctions. The signaling pathways that are upregulated by them the most are the MAPK (mitogen-activated protein kinases) signaling pathway, axon guidance, and the Ras signaling pathway. They also upregulate the AMPK, PI3K-Akt, Hippo, and Wnt signaling pathways and autophagy, though at a lower level than the first three mentioned [22].

The most upregulated miRNA found, miR-6741-3p, is highly likely to be responsible for combining the 3′-UTR of *UBQLN1*, a gene coding ubiquilin-1, a protein participating in the process of ubiquitination in proteasome-dependent protein degradation, via ubiquitin ligases [66]. An enzyme from this family, HERC3, was established to attenuate nuclear factor-κB-dependent signaling, which mediates inflammatory and immune reactions by inducing its ubiquitination and proteasomal degradation [67]. It means that the upregulation of miR-6741-3p results in lowering the inactivation level of NF-κB and increasing its activity, and consequently, in enhancing local and systemic inflammation, which are prominent in hemorrhoids [22,68].

Some of the upregulated miRNAs in hemorrhoidal extracellular vesicles target the MAPK (mitogen-activated protein kinases) signaling pathway, which is active in physiological and pathological cell proliferation, carcinogenesis [69] and angiogenesis [70,71] and may enhance inflammation as well [72]. The obtained results suggest that the MAPK pathway may be one of the factors increasing angiogenesis and partially increasing inflammation in hemorrhoids.

The number of downregulated miRNAs found in extracellular vesicles from hemorrhoidal tissues was 202, of which the 10 most significant were miR-548t-5p, miR-323b-5p, miR-1322, miR-3928-5p, miR-346, miR-4704-5p, miR-1913, miR-876-3p, miR-4460, and miR-892a. These are the molecules that possibly modulate transcriptional activator activity, also by regulating transcription, and they are the most active around the plasma membrane. The pathways targeted by them are mostly the Rap1 signaling pathway, and the synthesis, secretion, and action of the parathyroid hormone, proteoglycans in cancer, as well as, to a lesser extent, the MAPK, cAMP, and Wnt signaling pathways.

The expression pattern of miRNA may vary between proper hemorrhoidal tissues and extracellular vesicles emerging from them, since the same miRNAs are not always present at the same levels between them [22].

## 5. Nitric Oxide and Varicose Veins

Nitric oxide (NO) is a molecule produced in many different cells, and it contributes to vascular dilatation and the development of varicose veins [73]. It was found to be overly present, together with two Nitric Oxide Synthases (NOS), endothelial (eNOS) and neuronal (nNOS) [74] in hemorrhoids, while asymmetric dimethylarginine, a molecule that inhibits NOS, was underexpressed in those tissues [5,8,9,10,11,12,27,75,76]. The high expression of NOS in hemorrhoids leads to excessive amounts of NO being synthesized, and that causes an increase in blood flow and the twisting of veins, which adds to the varicose veins theory of the pathogenesis of hemorrhoids.

## 6. Angiogenesis

Hemorrhoids occur with increased angiogenesis, which leads to a high vascular density in the pathological tissue, the dilatation of blood vessels (which in the end might contribute to the development of varicose veins), and signs of edema, sometimes with thrombosis [5,8,9,10,11,12,27,76]. In some works, it was suggested that thrombosis might be one of many factors inducing angiogenesis (neovascularization) [77,78], possibly via the increase in the expression of VEGF [79,80], and that might be the case in hemorrhoids as well [76]. Nuclei of endothelial cells appear enlarged, and vascular endothelial cell markers vWF, CD31, and CD34, as well as endoglin, are expressed much more in hemorrhoids than in normal tissues, which further shows that there is intensified vascular proliferation present [26,76]. Endoglin is a glycoprotein overexpressed in endothelial cells, an accessory receptor for TGF-β, and a marker for angiogenesis, due to its appearance in different tissues with increased vascular proliferation [81,82,83,84]. Prominent levels of VEGF and VEGFR2, proangiogenic factors, were also identified in hemorrhoids, and they intensify the angiogenesis process as well [76,85].

On top of that, the signaling pathways responsible for inhibiting angiogenesis were found to be significantly less active in hemorrhoid cells, leading to increased vascular proliferation [26,27].

MiR-143-3p was significantly downregulated in hemorrhoid tissues, especially in severe cases. Its downregulation increases the expression of vascular markers, such as CD31, von Willebrand factor, and vascular endothelial growth factor receptor 2 (VEGFR2), and cell proliferation and migration, and enhances apoptosis, and it is a potential key regulator in angiogenesis in hemorrhoid progression and postoperative wound healing [86].

On chromosome 14, a genomic imprinted region is located—DLK1-DIO3 [39,40,41,42]. It contains, among others, microRNAs. Four of those miRNAs, miR-412-5p, miR-422-5p, miR-432-5p, and miR-1185-1-3p, were found to be expressed differently in healthy and hemorrhoidal tissues, and, in particular, miR-412-5p and miR-1185-1-3p were found to be significantly underexpressed in hemorrhoidal tissue [27].

**Figure 1:**
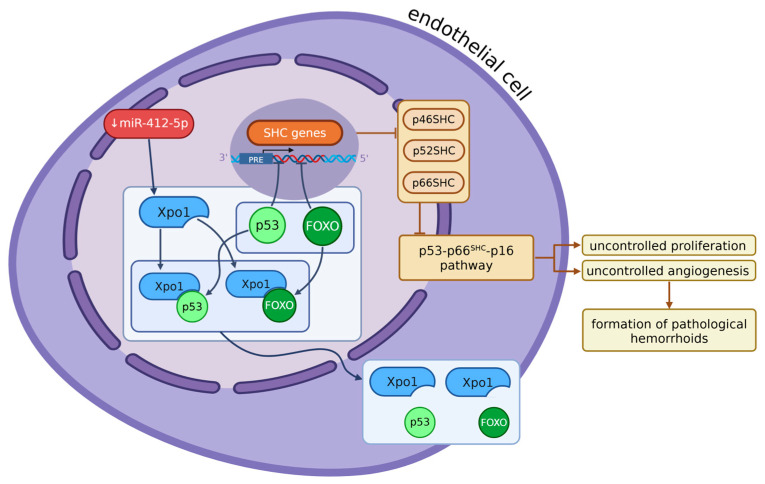
When miR-412-5p is underexpressed, Xpo1 is upregulated, which results in the excessive exporting of p53 and FOXO from the nucleus. The lack of p53 and FOXO in the nucleus reduces the expression of SHC genes—p46^SHC^, p52^SHC^, and p66^SHC^, which inhibits the p53–p66^SHC^–p16 pathway. This leads to uncontrolled proliferation and angiogenesis, contributing to the formation of pathological hemorrhoids (Xpo1—Exportin-1, p53—tumor suppressor protein, FOXO—Forkhead box O transcription factor, SHC—Src homology 2 domain-containing (p46^SHC^, p52^SHC^, p66^SHC^), p16—cyclin-dependent kinase inhibitor involved in cell cycle arrest). Created in BioRender. Mazurek, M. (2025) https://BioRender.com/c8w1axi.

MiR-412-5p targets the gene of Exportin1 (Xpo1) (see Figure 1) [27], a nuclear protein in endothelial cells that is responsible for exporting proteins, such as p53, p21, FOXO, PI3K/AKT, Wnt/β-catenin, AP-1, and NF-κB, from the nucleus to the cytoplasm [79,80,87,88]. The underexpression of miR-412-5p leads to the overexpression of Xpo1 in hemorrhoidal endothelial cells [27], since miRNAs silence gene expression and are the reason for the augmented translocation of p53 to the cytoplasm. The p53 protein in the nucleus is responsible for regulating the cell cycle by increasing the expression of certain genes. In the case of endothelial cells in hemorrhoids, those genes are the SHC genes. They are divided after transcription into three subtypes: p46^SHC^, p52^SHC,^ and p66^SHC^ [89,90,91]. The p66^SHC^ has a role in the Ras signaling pathway [89,90,92,93], inducing apoptosis and aging [89,90,91,94,95,96,97,98]. A lack of p53 in the nucleus of hemorrhoidal endothelial cells results in a decrease in SHC expression, especially p66^SHC^, blocking the p53-p66^SHC^-p16 pathway. The exact connection between that pathway and hemorrhoids is not clear yet, but it appears to inhibit and aggravate the cell cycle control, causing the proliferation of endothelial cells and angiogenesis, which is a factor in the formation of pathological hemorrhoids [27].

**Figure 2:**
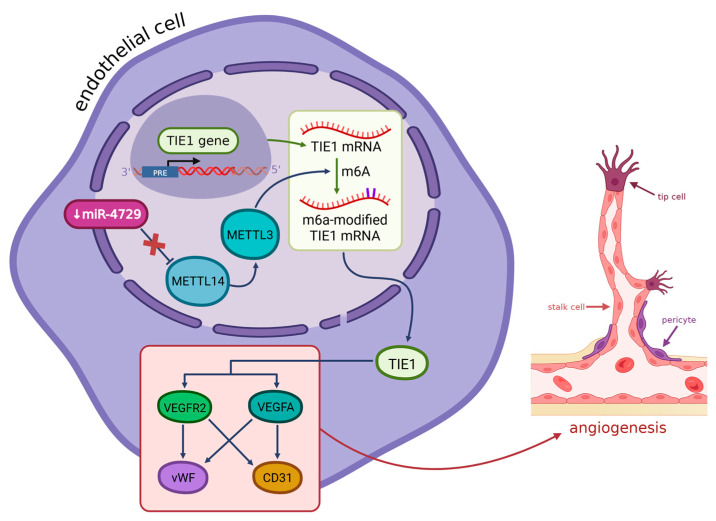
In hemorrhoidal vascular endothelial cells, miR-4729 is significantly downregulated, leading to the overexpression of METTL14, a key m6A RNA methyltransferase. Elevated METTL14 increases m6A methylation via activating METTL3 at the 3′ UTR of, enhancing TIE1 mRNA stability and translation. As a result, the TIE1 protein is overexpressed and activates the TIE1/VEGFA signaling loop, involving VEGFA, VEGFR2, vWF, and CD31, which promotes endothelial cell proliferation and angiogenesis, prominent in hemorrhoids (METTL3/14—methyltransferase-like 3/14, m6A—N6-methyladenosine, TIE1—tyrosine kinase receptor, VEGFA—vascular endothelial growth factor, VEGFR2—vascular endothelial growth factor receptor 2, vWF—von Willebrand factor, CD31—platelet endothelial cell adhesion molecule, tip/stalk cells—specialized endothelial cells involved in new vessel formation). Created in BioRender. Mazurek, M. (2025) https://BioRender.com/fcihnmu.

Another factor contributing to angiogenesis in hemorrhoids is changes in the methylation of RNA N-6 methyladenosine (m6ARNA) (see Figure 2). Enzymes from the m6A methyltransferase complex, described also as m6A writers, METTL14 and METTL3, form a heterodimeric complex, with METTL14 acting as a switch for METTL3’s activity, and they catalyze the methylation of m6A RNA [28,99,100,101,102]. Other components in that complex are auxiliary cofactors such as WTAP, VIRMA, RBM15/15B, ZC3H13, and HAKAI, which help with binding to the target RNA (first three), achieving the correct localization of the whole enzymatic complex (ZC3H1) [28]. For the further fate and function of m6A RNA, the readers are responsible: they improve its stability, regulate splicing, increase its transcriptional and translational activity, and promote carcinogenesis and invasion as well [28,100,102,103,104,105].

Ten miRNAs are predicted to target METTL14 expression, and from these, only one, miR-4729, was significantly low in hemorrhoid tissues. This miRNA, when it is upregulated, inhibits METTL14 expression in endothelial cells, and, as a result, inhibits m6A RNA methylation as well. It was later observed that in this case, proliferation, as well as overall vascular endothelial cell function, were inhibited. One gene was found to be especially inhibited in cells with miR-4729 overexpression—TIE1. TIE1 is a tyrosine kinase that plays a critical role in angiogenesis and blood vessel stability, as well as tissue remodeling and inflammation; it can be found in endothelial cells [106,107,108,109,110,111]. This gene is related to VEGFA, VEGFR2, vWF, and CD31 and can induce vWF and CD31 expression by activating VEGFA/VEGFR2 receptor ligands, which promotes angiogenesis. Together they form a signaling pathway called the TIE1/VEGFA signal molecular loop, in addition to the previously known TIE1/TIE2/VEGFR2 signaling pathway. TIE1 was found to be significantly underexpressed in cells with a high miR-4729 content, which suggests that this miRNA is a key factor regulating TIE1 expression, via influencing METTL14 expression, and then, as a result, m6A RNA methylation. In hemorrhoids, miR-4729 is underexpressed, which causes an increase in the synthesis of METTL14 and then an intensification in the methylation of m6ARNA, especially TIE1, which was found to be present in those cells at very high levels [26]. As a result, we can observe vascular hyperplasia, together with an increased expression of endothelial markers such as CD31, which is distinct in hemorrhoids.

## 7. A Role of Estrogen in Angiogenesis?

**Figure 3:**
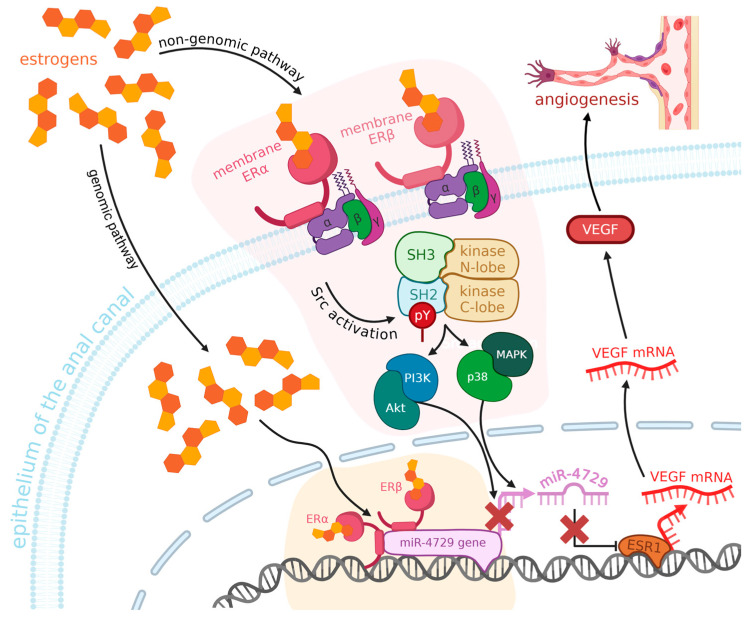
Estrogens activate ERα in the anal canal epithelium of women with hemorrhoids via genomic and non-genomic pathways, leading to Src activation and downstream signaling through PI3K/Akt, MAPK, and p38. This downregulates miR-4729, which results in the overexpression of ESR1 and enhances VEGF transcription. Increased VEGF promotes angiogenesis and edema in hemorrhoidal tissue (ERα—estrogen receptor alpha, ERβ—estrogen receptor beta, ESR1—estrogen receptor 1 gene, VEGF—vascular endothelial growth factor, PI3K—phosphoinositide 3-kinase, MAPK—mitogen-activated protein kinase, miR-4729—microRNA-4729, SH2/SH3—Src homology 2/3 domains). Created in BioRender. Mazurek, M. (2025) https://BioRender.com/158e9db.

In some cases of hemorrhoids in women, we can detect the presence of estrogen nuclear receptors (ERα) in the tissues of the anal canal [112,113]. They bind estrogens, which activate a signaling pathway leading to the downregulation of miR-424-5p. The higher the level of estrogens, the more ERα are present [114]. Low levels of miR-424-5p and elevated levels of ERα cause the upregulation of VEGF, and, as a result, angiogenesis and edema (see Figure 3). The opposite—a decrease in the expression of VEGF—happens when miR-424-5p is overexpressed. This miRNA’s predicted target is the ESR1 gene, whose product regulates the transcription of estrogen-inducible genes [115], such as *VEGF* [116]. The downregulation of miR-424-5p in hemorrhoids, caused by estrogens activating the ERα, leads to an increase in levels of ESR1 and then, as a result, VEGF, which promotes angiogenesis and edema in hemorrhoids in women. There is also some evidence of the role of estrogens in angiogenesis in other tissues—multiple studies across the years have reported that they are involved in the recovery of arterial endothelium after damage [117], possibly via promoting VEGF expression, as it is another function of estrogens, not only in hemorrhoids, but also in other tissues, both uterine and vascular [118,119], suggesting that their influence on hemorrhoids is not a solitary case.

## 8. Degeneration of Connective Tissue

The stroma of hemorrhoids consists of connective tissue, with collagen and elastic fibers cross-linked with each other, providing tensile strength, elasticity, integrity, and stability to the tissue; a layer of smooth muscle (Treitz’s muscle); and blood vessels [2,120].

Abnormalities in collagen structure, especially collagen I and III, participate in the development of hemorrhoids—whether due to its degradation or incorrect structure, which leads to poor mechanical endurance and stability. Conducted studies have shown that patients with hemorrhoids had many more defects in collagen structure than healthy patients, even those advanced in age. The same studies reported that collagen levels and collagen type I/III ratio were significantly reduced in hemorrhoids in comparison to normal anal cushions, with a significant predominance of collagen III [121], which results in less cross-linking and poor mechanical endurance, since the mature collagen I provides a greater tensile strength than the less mature collagen III. This suggests that the occurrence of hemorrhoids is associated with early and severe collagen degradation, as well as alterations in collagen type ratio. These changes may result from genetic, metabolic, or environmental factors, potentially leading to the early onset of the disease. However, the precise background of these alterations remains unclear and warrants further study [122,123].

Changes in the expression of elastic connective tissue components have been reported as well, in the fibulin family of extracellular matrix proteins. Fibulin-3 and fibulin-5 were found to be underexpressed in hemorrhoids [49,124,125,126]. Fibulin-5 stabilizes and organizes elastic fibers in extracellular matter and inhibits angiogenesis [127], whereas fibulin-3 maintains the stability of the basal membrane, stabilizes the extracellular matrix [128] and inhibits matrix metalloproteinases (MMPs)—hydrolytic enzymes responsible for the degradation of the extracellular matrix [129]. Low levels of those fibulins may result in damage to elastic fibers, and consequently, to the structure of connective tissue, predisposing the person to hemorrhoids, since the components of that tissue are linked to one another. Some medicines are successful in increasing their expression [49]. This suggests that some epigenetic mechanism may be regulating that expression, although the specific pathways are yet to be determined.

The levels of various MMPs have been found to be elevated in patients with hemorrhoids [21,22,49,130,131,132]. In grades I and II of the disease (early stages), those enzymes were MMP-1, MMP-2, and MMP-3; in grade III, MMP-3, MMP-7, MMP-8, and MMP-9; and in grade IV, MMP-2, MMP-8, and MMP-9 [21,133], with a very high expression of MMP-9, together with NGAL (neutrophil gelatinase-associated lipocalin, lipocalin-2)—a marker for neutrophil activation, and consequently for inflammation. NGAL and MMP-9 levels are particularly elevated in thrombosed and prolapsed hemorrhoids [21]. In addition to elastic fibers, these enzymes can target and degrade other components of the extracellular matrix, such as collagen. This process leads to inflammation and severe damage to the supportive tissue of the anal canal, as well as the disruption of regenerative processes. Consequently, it contributes to the progression of hemorrhoidal disease, particularly by promoting the development of prolapsed hemorrhoids, as the damaged supportive tissue is no longer able to prevent the prolapse of anal structures [49,131,132,133]. MMP-9 plays an additional role—it promotes angiogenesis by damaging the basal lamina of the endothelium, which stimulates the release of VEGF [134,135]. Prominent levels of those enzymes might be a consequence of the underexpression of fibulin-3 in hemorrhoidal tissues, as well as the activation of macrophages and lymphocytes, which secrete MMPs; however, that may not be the only mechanism. Further studies must be conducted to determine those mechanisms.

## 9. Genes Significant in the Development of Hemorrhoids

Along with the development of our knowledge about molecular biology, especially on the matter of genetics and epigenetics, the significant role of genes and changes associated with them as risk factors for hemorrhoidal disease has been widely considered. Numerous studies have been conducted across the span of years to research the involvement of genetics in the development of hemorrhoids and a possible genetic susceptibility to their occurrence, and we have aimed to gather and summarize them in our paper. The results are displayed in the table below (see Table 3). The mentioned genes and their mutations seem to be predisposing to hemorrhoids whether by impairing the function and structure of the epithelium and connective tissue, including the smooth muscles, changing the organization of the extracellular matter (ECM), limiting neuromuscular motility, as well as enhancing the synthesis of some proteins. Those changes can be and were previously associated with gastrointestinal, neuroaffective, and cardiovascular conditions [136].

## 10. Possible Epigenetic Factors in the Development of Hemorrhoids

**Figure 4:**
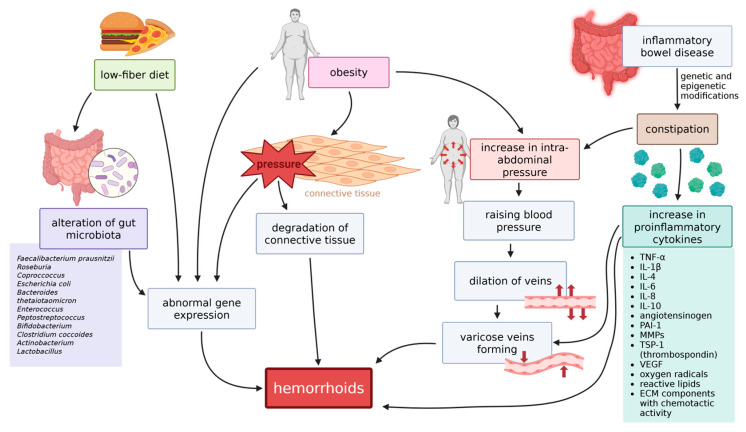
Hemorrhoid development is influenced by epigenetic and lifestyle factors. A low-fiber diet alters gut microbiota, affecting gene expression. Obesity increases intra-abdominal pressure and inflammation via cytokines, while constipation, present in IBS and IBD, raises venous pressure and degrades connective tissue, promoting varicose vein *formation* (TNF-α—tumor necrosis factor alpha, IL—interleukin, VEGF—vascular endothelial growth factor, IBS—irritable bowel syndrome, IBD—inflammatory bowel disease). Created in BioRender. Mazurek, M. (2025) https://BioRender.com/rnrh8xd.

There are numerous environmental and lifestyle-dependent factors considered to be connected to the pathogenesis of hemorrhoids. Some of those factors, discussed below, are either known or suspected to cause epigenetic changes in the cells, which can lead to changes that are a direct cause for hemorrhoids (see Figure 4).

### 10.1. Diet and Gut Microbiota

Dietary changes, such as a low-fiber diet, are known to change the composition of the gut microbiota. Although the change in microbiota components is not conditioned by epigenetic mechanisms, it can induce epigenetic modifications in the organism. Diet compounds, together with their bacterial metabolites, can influence gene expression via removing or providing compounds that are substrates for epigenetic modifications [171]. Among bacterial species that influence the levels of such proteins are butyrate-producing species such as *Faecalibacterium prausnitzii*, *Roseburia*, and *Coprococcus* species; species that can produce isothiocyanates, such as *Escherichia coli*, *Bacteroides thetaiotaomicron*, *Enterococcus*, *Peptostreptococcus*, and *Bifidobacterium*, or those that metabolize ellagitannins to urolithins—e.g., *Clostridium coccoides*, *Actinobacterium*, *Lactobacillus*, or—again—*Bifidobacterium* [172].

### 10.2. Obesity

Across many different studies, obesity has been named as a factor increasing the probability of developing hemorrhoids [173,174,175,176]. Aside from exerting pressure on abdominal tissues, adiposity also escalates the inflammatory process in tissues by stimulating the release of pro-inflammatory cytokines—among others adipokines, TNF-α, IL-6, and other interleukins—as well as angiotensinogen, PAI-1 (plasminogen activator inhibitor-1), MMPs, TSP-1 (thrombospondin), VEGF, oxygen radicals, reactive lipids, and extracellular matrix components with chemotactic activity [177,178,179], which accelerates inflammation [176].

### 10.3. Constipation and Irritable Bowel Syndrome

Constipation has been known as one of the crucial factors for hemorrhoids for a long time and is still being recognized in modern research [174,180,181,182]. There are various causes for constipation, and amongst them is irritable bowel syndrome (IBS)—a disorder of the brain–gut axis, resulting in abdominal pain and an alteration of the frequency of bowel movements. Recently, works hinting at the possible association of IBS with hemorrhoids have been published [136,183,184,185]. IBS is a multifactorial condition, and it is mediated, among others, by genetic and epigenetic mechanisms [186]. Hemorrhoids may be a result of genetic and epigenetic modifications in IBS. It is also theorized that changes in the gut microbiota, possibly caused by a low-fiber diet, can participate in inducing constipation [187].

## 11. Discussion

Aside from the well-recognized environmental and mechanical factors in the development of hemorrhoids, such as constipation, obesity, and straining, thanks to novel research we can distinguish the molecular pathways that participate in the pathogenesis of this condition (see Table 4). These molecular alterations may be triggered by environmental and mechanical factors long associated with the disease. For instance, hemorrhoids occurring in relation to IBS are associated with induced epigenetic modifications. Dietary changes and a low-fiber diet can both alter the gut microbiota and induce epigenetic modifications. The details of those mechanisms remain to be fully clarified [187]. Obesity is another well-established risk factor—aside from exerting pressure and mechanical stress on anal cushions, it also acts to induce systemic low-grade inflammation and the epigenetic regulation of gene expression, thereby promoting vascular changes and connective tissue degeneration. Such observations suggest that environmental and metabolic factors not only impose mechanical stress but may also influence molecular pathways, linking classical risk factors with emerging genetic and epigenetic mechanisms in hemorrhoidal disease.

One of the most consistent observations across studies is the dysregulation of inflammatory pathways and the altered expression of inflammatory pathways. Hemorrhoidal tissues show an upregulation of pro-inflammatory cytokines (e.g., TNF-α, IL-1β, IL-6, VEGF) and a concurrent downregulation of anti-inflammatory mediators. MiRNAs, such as miR-770, miR-143-3p, or miR-4729, modulate cytokine pathways, which suggests that targeting miRNA activity, either through analogs or inhibitors, could become a therapeutic approach.

Angiogenesis is another critical molecular pathway involved in hemorrhoidal disease development. Molecules such as VEGF and VEGFR2, as well as the altered activity of miRNAs (e.g., miR-412-5p, miR-1185-1-3p) and m6A RNA methylation enzymes (METTL14, METTL3) are the processes that fuel excessive vascular proliferation. The inhibition of these angiogenic pathways, already pursued in oncology and ophthalmology [188], could be adapted for hemorrhoids if safety and local delivery challenges are addressed. Moreover, the link between estrogen signaling and VEGF expression further highlights a possible sex-specific therapeutic approach, though this requires further validation.

Equally important is the degeneration of connective tissue—the evidence of this being altered collagen I/III ratios, the reduced expression of fibulins, and the excessive activity of MMPs. Our knowledge of the epigenetic regulation of fibulin expression and MMP secretion remains insufficiently detailed, but these pathways could represent potential points of intervention to restore the structural integrity of anal cushions and the surrounding tissues. (e.g., FDA-approved application in periodontal disease and investigational use in cancer, diabetic foot ulcers, and multiple sclerosis), may hold promise for stopping disease progression [189].

## 12. Conclusions

Although no clinical recommendations can be drawn from the currently available data, the accumulating evidence suggests several emerging molecular targets for the treatment of hemorrhoids: (i) the inhibition of pro-inflammatory cytokines, (ii) the modulation of key miRNAs, (iii) the regulation of angiogenic signaling (VEGF/VEGFR2, TIE1/VEGFA loops), (iv) the restoration of connective tissue homeostasis through the regulation of fibulins and MMPs. These results illustrate that hemorrhoids should be considered not merely a mechanical disorder but a disease with a strong molecular background, opening possibilities for targeted, molecular-based therapies. Future research should focus on the validation of miRNA-based interventions, as well as inhibitors of angiogenic pathways and modulators of extracellular matrix remodeling in hemorrhoids. Based on them and by shifting focus from symptomatic treatment to molecular foundations, more effective treatments for hemorrhoidal disease may be developed.

## 13. Materials and Methods

**Figure 5:**
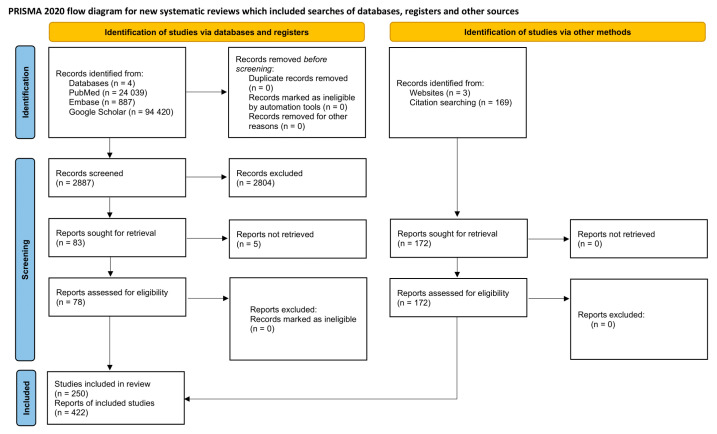
The diagram above demonstrates the process of gathering evidence while performing this review. Page MJ, et al. BMJ 2021;372:n71. doi: 10.1136/bmj.n71 [191].

Our narrative review was conducted following the SANRA (Scale for the Assessment of Narrative Review Articles) checklist [190], as it is the standard for narrative reviews. We aimed for our work to meet all six quality criteria of these guidelines to ensure the highest standards of scientific rigor and reliability, and we have used the PRISMA flow chart to visualize the process of conducting the review [191] (see Figure 5).

For the search, we used PubMed, Embase, and Google Scholar, as this combination of scientific databases was shown to be sufficient for reviews in peer-reviewed studies [192,193]. We searched for data about genetic and epigenetic factors contributing to the development of hemorrhoids. The search was conducted using a combination of keywords, one combination for one search: (“hemorrhoids”) AND (“genetics” OR “epigenetics” OR “genetic factors” OR “epigenetic factors” OR “factors”). Terms were searched as a combination of the word “hemorrhoids” and one word from the second group, e.g., “hemorrhoids genetics”. The initial search revealed the following number of records, combined from all keywords: *n* = 24,039 for PubMed, *n* = 887 for Embase, and *n* = 94,420 for Google Scholar, finding a total of 119,346 records. Based on the initial search, we have decided to limit the number of publications qualified for screening to 200 per search. This limited the number of results to 2887, from which we identified papers that met our criteria.

In the first screening, we looked for papers that mentioned hemorrhoids in the title, keywords, or abstract, together with genetics in a broad sense, gene expression, specific epigenetic mechanisms, or changes in mechanisms that are likely to be mediated by epigenetics. We tried to minimize errors by the double screening of papers by two independent researchers. Moreover, after identifying papers, to minimize errors, we utilized first-pass screening, including only titles and keywords, and second-pass screening, including abstracts; the full text was read during the second pass if needed.

Based on the criteria mentioned above, we identified, while also excluding the duplicates, 83 articles. The articles included in this study comprised only original papers about performed studies, meta-analyses, as well as reviews, which suited the topic of our review. At this point, all types of research papers other than meta-analyses, reviews, and original studies were excluded. We performed a secondary review of the articles we first identified, which led to the exclusion of five more articles due to their irrelevance to the topic of this study after reviewing their text, the lack of the full text in a language understandable to us, or inability to be retrieved. If a paper was deemed irrelevant by one author but relevant by another, it was discussed until a unanimous decision was reached. The inclusion criteria stayed the same as during the search for papers.

The next step in our research was citation searching. We searched previously identified papers and added more articles to our base. Finally, 250 articles were included for data extraction and analysis.

We divided the articles from our base into four sub-groups: those concerning only genetics, only epigenetics, possibly epigenetics, and a mixed sub-group, including both genetics and epigenetics. One part of our team was responsible for reviewing articles and writing the text related to the involvement of genetics in hemorrhoids, based on groups, genetics and mixed genetics, and epigenetics, while the other part focused solely on epigenetic mechanisms by reviewing articles from group epigenetics, possibly epigenetics, and mixed genetics and epigenetics.

## Figures and Tables

**Table 1:** Cytokines presented in the table were identified in hemorrhoidal tissues.

Cytokines Expressed in Hemorrhoids	
Pro-Inflammatory Cytokines	Anti-Inflammatory Cytokines
RANTES	IL-10
TNF-α	··
VEGF	··
IL-1β	··
IL-6	··
IL-8	··
IL-17	··
IFN-γ	··

**Table 2:** MiRNAs differently expressed in hemorrhoids seem to target genes that influence various vesicle pathways.

	MiRNAs in Vesicle Pathways in Hemorrhoids		MiRNAs in Extracellular Vesicle Pathways in Hemorrhoids	
**Probable targeted processes** *	Endocytosis	Synaptic vesicle pathways	Transcription, protein kinase activity, and ubiquitination	Transcriptional activator activity
**Status**	Upregulated	Downregulated	Upregulated	Downregulated
**Type of miRNA**	miR-375	miR-376b-3p	miR-6741-3p	miR-548t-5p
	miR-215-5p	miR-34a-5p	miR-6834-3	miR-323b-5p
	miR-192-5p	miR-152-3p	miR-425	miR-1322
	miR-143-3p	let-7c-5p	miR-6804-3	miR-3928-5p
	miR-187-3p	miR-107	miR-744-3	miR-346
	miR-194-5p	miR-517a-3p	miR-848	miR-4704-5p
	miR-145-5p	miR-517b-3p	miR-299-5	miR-1913
	miR-490-3p	miR-1307-5p	miR-463	miR-876-3p
	miR-145-3p	miR-190a-5p	miR-317	miR-4460
	··	miR-378a-5p	miR-465	miR-892a
	··	miR-708-3p	··	··
	··	miR-450a-5p	··	··
	··	miR-30e-5p	··	··
	··	miR-532-5p	··	··

* These are processes associated with genes that the listed miRNAs possibly target, according to available databases.

**Table 3:** Genes displayed in the table were proposed or reported to contribute to the pathogenesis of hemorrhoids (“—" applies to unknown, unavailable values).

Genes Significant to the Development of Hemorrhoids					
Gene	Product	Function	Mechanism of Action	Mutations	Reported Associated Conditions
*FOXC2*	FOXC2 (forkhead box protein) [137,138]	− development of venous cardiovascular and lymphatic system [139,140,141]	—	− 91 C-G transversion, in proximal upstream region of FOXC2 [142] − frameshift mutation of base pair insertion at 880’881insT in the coding region (results in premature ‘stop’ codon at bp 1386) [143]	− lymphedema distichiasis [144] − chronic venous disease [138,141,145] − varicose veins − 880’881insT possibly linked to hemorrhoids, varicose veins and myocardial infarction [143]
*NOX1* and *NOS3*	NOX1 (NADPH oxidase) NOS3 (nitric oxide synthase)	− fluid shear stress − abnormal wound healing − regulation of the cell − cofactor binding − components of the plasma membrane protein complex [146] − maintaining the intestinal crypt homeostasis and microbiota composition [147] − vasodilatation [74,148,149]	− generating reactive oxygen species (ROS), which damage DNA, proteins, and lipids − *ADMA* (asymmetric dimethylarginine—NOS inhibitor) is underexpressed in hemorrhoids [74]	—	− cardiovascular diseases and hypertension [150] − chronic functional constipation [151] − inflammatory bowel disease [147] − hemorrhoids [75,146] − atherosclerosis [146]
*MTHFR*	methylenetetrahydrofolate reductase	− metabolizes folate − controls homocysteine levels [152]	—	− polymorphism C677T (alanine to valine substitution in the N-terminal catalytic domain) − polymorphism A1298C (alanine to glutamine substitution in the C-terminal regulatory domain) [101] − polymorphism 677 [153]	− Polymorphisms C677T and A1298C were shown to predispose to CVD [154] − polymorphism 677, when accompanied by constipation, hemorrhoids, family history of rectal cancer and was linked to the risk of colorectal cancer [153] − venous thrombosis, no association with thrombosed hemorrhoidal disease was found [155]
*MYH9*	heavy chain of non-muscle myosin IIA	− angiogenesis [156]	—	− SNP rs735854 [157]	− mutations lead to thrombocytopenia and platelet macrocytosis; can be related to bleeding disorders [158] − varicose veins and hemorrhoids [157]
*F5*	coagulation factor V	− coagulation cascade [159]	—	− polymorphism rs6546324 − rs6025—missense mutation (p.Arg534Gln) [160]	− endometriosis − hemorrhoids [160] − venous thrombosis [159]
*CYP1A*	aryl hydrocarbon hydroxylase in hepatic and extrahepatic cytochrome P450	—	—	− polymorphism CYP1A1*2A (transition of thymidine to cytosine at position 3801 in the 3′UTR) − various single allele mutations [161]	− mutations are protective for hemorrhoids and peripheral circulatory problems [161]
*PON1*	serum paraoxonase 1	—	—	− Q isoform polymorphism (glutamine at position 192) − R isoform polymorphism has arginine [162,163] − leucine/methionine substitution at 54. position [164]	− Q/R isoform polymorphism has a protective effect on chronic constipation − leucine/methionine substitution is associated with a higher risk of hemorrhoids [161]
*CALM3*	calmodulin 3	− signal transduction − muscle contraction − enzyme regulation − pain − fever − constipation − inflammation − proliferation [165]	—	—	− increased expression in hemorrhoids − anemia, leukemia [166]
*ANO1*	anoctamin-1 (voltage-gated calcium-activated anion channel)	− control of intestinal motility and peristalsis	− p.Phe608Ser mutation leads to an increase in the Cl^−^ current and a slowdown of activation/deactivation of the channel − SNP rs2186797 destabilizes the protein structure	− SNP rs2186797 − amino acid change p.Phe608Ser [136]	—
*SPRX*	—	− located on chromosome X − component of extracellular matter (ECM), especially in colon and liver [167]	− rs35318931 may destabilize the C-terminal domain of the amino acid chain	− missense mutation rs35318931 − amino acid change p.Ser413Phe [136]	—
*ACHE*	acetylcholinesterase	− hydrolyzation of acetylcholine at neuromuscular junctions	—	− mutation rs4556017	− increased expression in hemorrhoids [84] − Hirschsprung’s disease [168]
*SRTT*	capped-RNA binding protein	—	—	—	− increased expression in hemorrhoids [136]
*GSDMC*	gasdermin C	− expressed in epithelial cells	—	− mutation rs10956488	− increased expression in hemorrhoids [136]
*MYH11*	muscle myosin heavy chain 11	− ECM organization − muscle contraction	− overexpression linked to increased autophagy and impairment of contractile signaling, leading to a decrease in protein levels [169]	− mutation rs6498573	− increased expression in hemorrhoids [136]
*ELN*	elastin	− component in the extracellular matrix	—	—	− increased expression in hemorrhoids − cutis laxa [136]
*COL5A2*	type V collagen (regulatory)	—	—	—	− increased expression in hemorrhoids [136] − Ehler–Danlos syndrome (hypothesized to be linked to hemorrhoids) [170]
*PRDM*	histone methyltransferase	− regulation of gene expression − affects vascular smooth muscle cells contractility	—	—	− increased expression in hemorrhoids [136]

**Table 4:** A summary table presenting molecular factors involved in the development of hemorrhoids.

Molecular Components Altered in Hemorrhoids			
Type of Change	Upregulated	Downregulated	Mutated or Altered (*) Genes
**Molecule**	Pro-inflammatory cytokines, especially VEGF	Anti-inflammatory cytokines	*FOXC2*
	CGRP, SP, and TRPV1	Inhibitory pathways of angiogenesis	*MTHFR*
	COX-2	Vesicle miRNAs modulating synaptic vesicle pathways and transcriptional activator activity	*MYH9*
	Vesicle miRNAs modulating endocytosis, transcription, protein kinase activity, and ubiquitination	miR-412-5p, leading to deregulation of the cell cycle	*CYP1A*
	NOS3	miR-4729, leading to overexpression of *TIE1* and angiogenesis via *METTL14* regulation	*PON1*
	NOS and NO	miR-424-5p, caused by estrogens, leading to increased expression of *VEGF*	*ANO1*
	vWF, CD31, CD34, endoglin	Fibulin-3 and fibulin-5	*SPRX* *
	VEGF, VEGFR2	Collagen levels, collagen I/III ratio	*SRTT* *
	MMPs	··	*GSDMC* *
	NOX1	··	*COL5A2*
	CALM3	··	··
	ACHE	··	··
	MYH11	··	··
	ELN	··	··
	PRDM	··	··

* The phrase “altered genes” refers to genes that were found to be altered in hemorrhoidal cells, in comparison to normal cells, but the nature of that change is not specified.

## Abbreviations

The following abbreviations are used in this manuscript:

FOXC2	Forkhead Box C2
NOX	NADPH Oxidase
NOS	Nitric Oxide Synthase
CALM3	Calmodulin 3
miRNA	microRNA
mRNA	Messenger RNA
DNA	Deoxyribonucleic Acid
COX2	Cyclooxygenase-2
RYBP	Ring1 and YY1-binding protein
PRC1	Polycomb Repressive Complex 1
NF-κB	Nuclear Factor Kappa-light-chain-enhancer of Activated B Cells
PGE2	Prostaglandin E2
ncRNA	Non-coding RNA
lncRNA	Long non-coding RNA
rRNA	Ribosomal RNA
tRNA	Transfer RNA
snRNA	Small nuclear RNA
UTR	Untranslated Region
MMP	Matrix Metalloproteinase
RANTES	Regulated on Activation, Normal T-cell Expressed and Secreted
CCL-5	Chemokine (C-C motif) Ligand 5
TNF-α	Tumor Necrosis Factor Alpha
VEGF	Vascular Endothelial Growth Factor
IL	Interleukin
IFN-γ	Interferon Gamma
CGRP	Calcitonin Gene-Related Peptide
SP	Substance P
TRPV1	Transient Receptor Potential Vanilloid 1
MAPK	Mitogen-Activated Protein Kinase
AMPK	AMP-Activated Protein Kinase
PI3K	Phosphoinositide 3-Kinase
UBQLN1	Ubiquilin-1
HERC3	HECT and RLD Domain Containing E3 Ubiquitin Protein Ligase 3
ECM	Extracellular Matrix
vWF	von Willebrand Factor
CD	Cluster of Differentiation
TGF-β	Transforming Growth Factor Beta
Xpo1	Exportin 1
PI3K/AKT	Phosphoinositide 3-Kinase/Protein Kinase B
AP-1	Activator Protein 1
SHC	Src Homology 2 Domain-Containing
m6A	N6-methyladenosine
METTL14	Methyltransferase-Like 14
METTL3	Methyltransferase-Like 3
WTAP	Wilms Tumor 1-Associated Protein
VIRMA	Vir-Like m6A Methyltransferase-Associated
RBM15/15B	RNA Binding Motif Protein 15/15B
ZC3H13	Zinc Finger CCCH-Type Containing 13
TIE1	Tyrosine Kinase with Immunoglobulin-Like and EGF-Like Domains 1
VEGFA	Vascular Endothelial Growth Factor A
ESR1	Estrogen Receptor 1
ERα	Estrogen Receptor Alpha
IBS	Irritable Bowel Syndrome
PAI-1	Plasminogen Activator Inhibitor-1
TSP-1	Thrombospondin-1
NGAL	Neutrophil Gelatinase-Associated Lipocalin
SANRA	Scale for the Assessment of Narrative Review Articles
